# Plasma orexin A does not reflect severity of illness in the intensive care units patients with systemic inflammation

**DOI:** 10.1186/s40981-022-00498-4

**Published:** 2022-01-22

**Authors:** Masahiro Akaishi, Eiji Hashiba, Daiki Takekawa, Tetsuya Kushikata, Kazuyoshi Hirota

**Affiliations:** Department of Anesthesiology, Hirosaki University Graduate School of Medicine, Hirosaki, 036-8562 Japan

**Keywords:** Orexin, Procalcitonin, Presepsin, Neutrophil gelatinase-associated lipocalin, Severity of Illness, Inflammation, Biomarkers

## Abstract

**Background:**

Systemic inflammatory response occurs by sepsis and invasive surgery. Recent articles suggest that not only CRP but also procalcitonin, presepsin, and neutrophil gelatinase-associated lipocalin may reflect the severity of systemic inflammation. In addition, as systemic inflammation could degenerate orexin neurons, plasma orexin A might also be a good biomarker to predict the severity. Thus, we have determined relation between plasma biomarker and severity of illness score in patients with systemic inflammation.

**Methods:**

Previous database (UMIN000018427) was used to secondly determine which plasma biomarkers may predict the severity of illness in the ICU patients with systemic inflammation (*n* = 57, 31 non-sepsis surgical patients and 26 sepsis patients). We measured plasma levels of orexin A, CRP, procalcitonin, presepsin, and neutrophil gelatinase-associated lipocalin were measured, and APACHE II score was assessed in these patients at their admission to the ICU. Data are shown as mean ± SD. Statistical analyses were done with unpaired *t* test. The correlation between APACHE II score and plasma biomarkers were examined using Pearson’s correlation coefficient and a least squares linear regression line.

**Results:**

Demographic data did not differ between sepsis and non-sepsis groups. However, APACHE-II score was significantly higher in sepsis group than those in non-sepsis group (20.9 ± 6.6 vs 15.8 ± 3.2, *p* < 0.01). There were significant correlations between APACHE II score and plasma CRP (*r* = 0.532, *p* < 0.01), procalcitonin (*r* = 0.551, *p* < 0.01), presepsin (*r* = 0.510, *p* < 0.01), and neutrophil gelatinase-associated lipocalin (*r* = 0.466, *P* < 0.01) except orexin A.

**Conclusion:**

All plasma biomarkers tested except orexin A may reflect the severity of illness in patients with systemic inflammation.

## Introduction

It is well known that systemic inflammation is induced by sepsis [1] and extensive cancer surgeries [2]. Recent articles [3–5] suggest that not only CRP but also procalcitonin, presepsin and neutrophil gelatinase-associated lipocalin (NGAL) may reflect the severity of SIRS. In addition, recent animal studies showed that inflammation degenerate orexin (OX) neurons and reduce OXergic activity [6–8]. Indeed, we found that lipopolysaccharide (LPS) significantly reduces OXA content in the pons and there was a good correlation between OXA content in the pons and survival rate of rats [9]. We previously suggested that plasma OXA may reflect neuronal OXergic activity because of significant increases in plasma OXA at emergence from general anesthesia.

Thus, we hypothesize that plasma OXA might be a good biomarker to predict the severity of illness in patients with systemic inflammation.

## Methods

### Clinical assessment

We secondly analyzed previous our clinical study data (UMIN000018427) that was presented in 31st Annual Congress of European Society of Intensive Care Medicine [10]. The study period was between 1 October 2015 and 28 February 2017. In this period, we enrolled 31 non-sepsis and 26 sepsis patients admitted to the intensive care unit (ICU). Then, we assessed APACHE II score of each patient.

### Measurement of biomarkers

Blood was collected from all patients to measured plasma biomarkers such as OXA, C-reactive protein (CRP), procalcitonin, presepsin and NGAL at the ICU admission. Plasma CRP, procalcitonin, and presepsisin were measured in Clinical Laboratory Department in our hospital. Plasma CRP was measured using JCA-BM6070 Biomajesty (JEOL, Tokyo) and the lower limit of detection was 0.02 mg/dL. Plasma presepsin were determined with a compact automated immunoanalyzer (PATHFAST; Mitsubishi Chemical Medience Corporation, Tokyo, Japan) using a chemiluminescent enzyme immunoassay. The lower limit of detection was 20 pg/mL. Plasma procalcitonin were measured via an electrochemiluminescent immunoassay using Elecsys reagents, the Elecsys BRAHMS PCT, and a Cobas e411 (Roche Diagnostics, Tokyo, Japan), according to the manufacturer’s instructions. The lower limit of detection was 0.02 ng/mL. Plasma OXA and NGAL were quantified using ELISA kits (OXA: Peninsula Laboratories International, San Carlos, CA, USA, NGAL: BioPorto Diagnostics A/S, Demark) in our departmental laboratory. The orexin A and NGAL assays were carried out in duplicate and mean intra- and inter-assay coefficient of variation were 5% and 10% and 14% and 14%, respectively.

### Statistics

Statistical analyses were done with unpaired t-test. Correlation between biomarkers and also between each biomarker and APACHEII scores were determined with Pearson’s correlation coefficient from a least square’s linear regression line using GraphPad Prism V3 (GraphPad Software Inc., CA, USA). Post-hoc power analyses of the correlations were performed using G* power version 3.1 [11].

## Results

There are no significant differences in patients’ background between sepsis and non-sepsis groups except underlying diseases although APACHEII score were significantly higher in sepsis group than those in non-sepsis group (Table 1).

**Table 1:** Patients’ background

	Sepsis (*n* = 26)	Non-sepsis (*n* = 31)
Sex (male/female)	12/14	20/11
Height (cm)	154.5 ± 7.2	159.1 ± 7.1
Weight (kg)	55.5 ± 11.5	50.2 ± 10.2
Age (years)	76.1 ± 10.1	67.7 ± 10.6
Length of ICU stay (days)	6. 9 ± 7.7	4.2 ± 2.4
Length of hospital stay (days)	38.2 ± 41.0	47.2 ± 21.5
APACHEII score	20.9 ± 6.6	15.8 ± 3.2*
Underlying diseases	Peritonitis (*n* = 10)	Oral ca (*n* = 20)
	UTI (*n* = 9)	Esophageal ca (*n* = 11)
	Others (*n* = 7)	

Mean ± SD, *: *p* < 0.01. *UTI* urinary tract infection, *ca* cancer

**Fig. 1:**
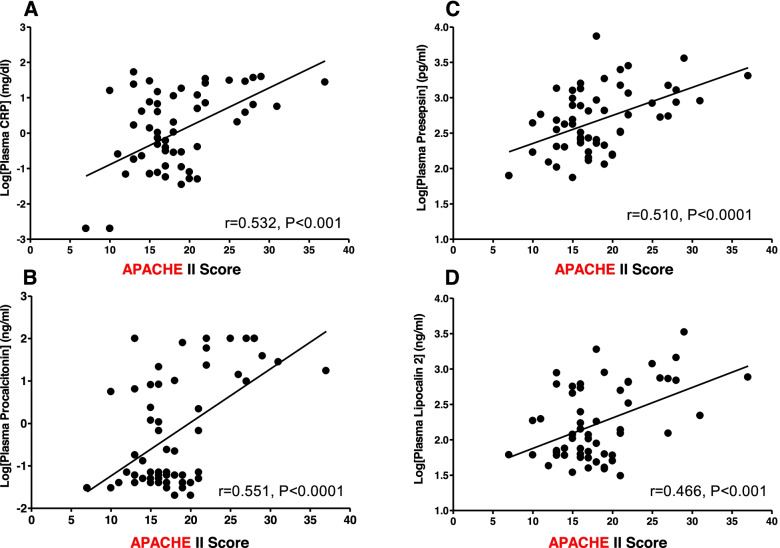
There was significant correlation between APACHE II and plasma inflammatory biomarkers. **A** CRP. **B** Procalcitonin. **C** Presepsin. **D** NGAL (neutrophil gelatinase-associated lipocalin)

**Fig. 2:**
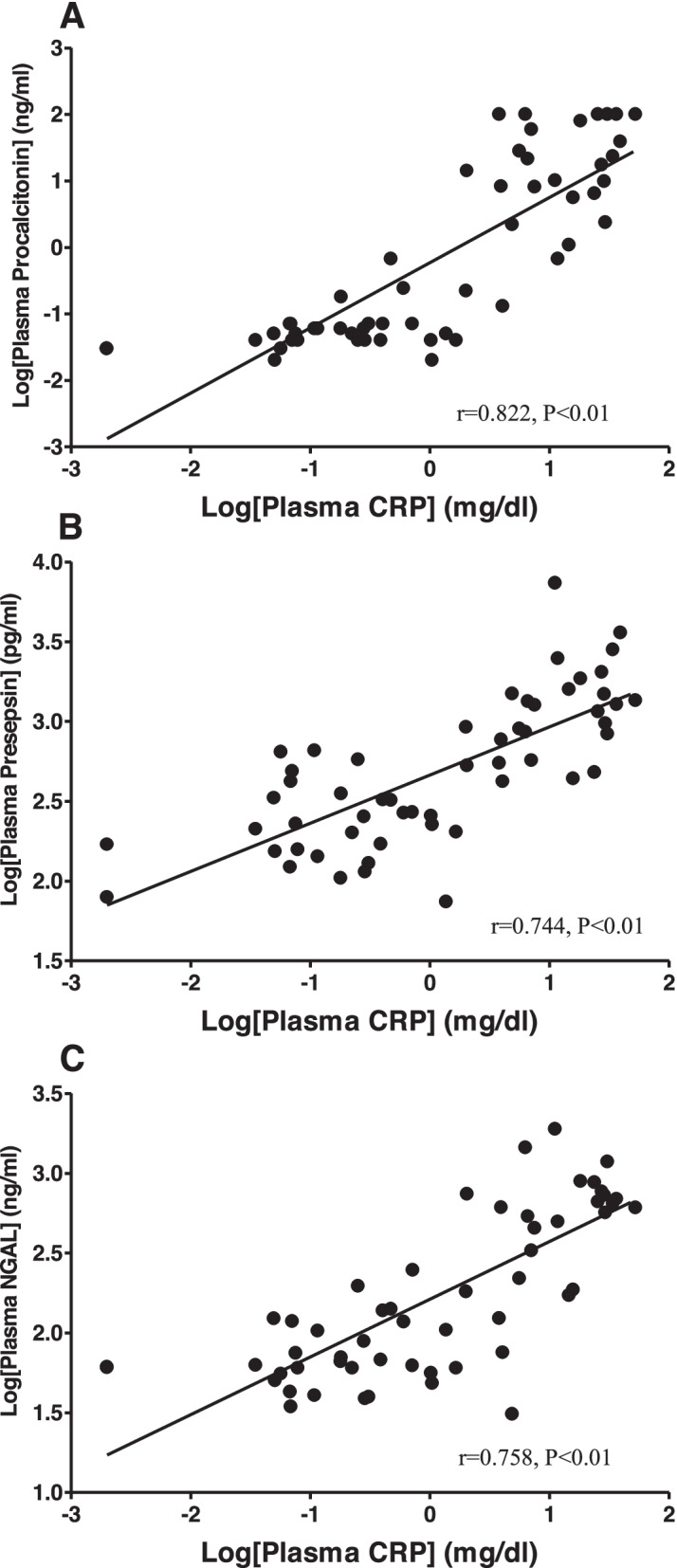
There was significant correlation between CRP and other plasma inflammatory biomarkers. **A** Procalcitonin. **B** Presepsin. **C** NGAL (neutrophil gelatinase-associated lipocalin)

In addition, there were significant correlations between APACHEII score and plasma inflammatory biomarkers such as CRP, presepsin, procalcitonin and NGAL (Fig. 1) except OXA. In addition, plasma CRP was significantly correlated to presepsin, procalcitonin, and NGAL (Fig. 2). However, there was no correlation between OXA and APACHEII (*r* = 0.229, *p* = 0.0864) or CRP (*r* = 0.056, *p* = 0.6941). The results of post-hoc power analyses of the correlations were more than 96% except those with OXA.

## Discussion

### Inflammatory biomarkers and severity of illness

In the present study, we found that inflammatory biomarkers such as CRP, procalcitonin, presepsin, and NGAL are significantly correlated with APACHE II score. Similarly, Hu and colleagues [3] found significant correlation between plasma biomarkers: pentraxin-3, procalcitonin, and lactate and severity of illness scores: SOFA and APACHE II scores. Takahashi and colleagues [4] reported significant association of NGAL, cystatin C, and estimated glomerular filtration rate with procalcitonin, presepsin and APACHE II score, and also significant correlation of both procalcitonin and presepsin with APACHE II score in infectious patients with acute kidney injury. Szederjesi et al. [5] also reported significant positive association between procalcitonin and all severity scores assessed (APACHEII, SOFA and SAPS II) and between CRP and only SAPS II score. Thus, inflammatory biomarkers may be clinically informative to assess disease severity of ICU patients.

### Orexin and severity of illness

The OXergic neurons could degenerate by neuroinflammation with the decline of OXergic activity, which may partly characterize the symptoms of sepsis [12]. Indeed, we recently found that LPS significantly reduced OXA contents in the pons containing the locus coeruleus which may impair sympathetic nervous system [9]. Several reports [13, 14] suggest that plasma OXA may reflect OXergic activity. Patients with severe obstructive sleep apnea syndrome have been reported to show a significant association between plasma OXA and the arousal index [13]. Plasma OXA has also been correlated with cerebrospinal fluid (CSF) OXA in the healthy subjects [14]. We also reported that plasma OXA may reflect OXergic activity at emergence from general anesthesia [15, 16]. However, the present study showed no correlation between plasma OXA and both APACHE II score and CRP although the other inflammatory biomarkers significantly correlated with APACH II score. Although the post-hoc power analyses of the correlations related to OXA were much less than 80%, the correlation coefficients were quite low. It was suggested that plasma OXA would not have sensitively indicated severity of illness and grade of inflammation in patients even if the numbers of patients were increased in the study. Plasma levels of OXA did not significantly decrease in septic patients in our previous study [10]. The reason why there was a discrepancy between the clinical study and animal study was unknown so far. Kotan and colleagues [17] found an inverse correlation between the CSF OXA and the infarct volume in patients with ischemic stroke but no significant correlation between the serum OXA and the infarct volume This report suggests that plasma OXA may not always reflect central OXergic activity. Although the levels of CSF OXA or the central OXergic activities were not measured in this study, plasma OXA may not be an appropriate biomarker to assess severity of illness in the ICU.

## Conclusion

The present data suggest that plasma CRP, presepsin, procalcitonin, and NGAL may be good biomarkers to assess severity of illness in the ICU patients with systemic inflammation. However, plasma OXA may not reflect the severity of illness.

## Data Availability

The datasets used and/or analyzed during the current study are available from the corresponding author on reasonable request.
